# Molecular Mechanisms Underlying Detection Sensitivity in Nanoparticle-Assisted NMR Chemosensing

**DOI:** 10.1021/acs.jpclett.3c01005

**Published:** 2023-07-27

**Authors:** Sebastian Franco-Ulloa, Andrea Cesari, Laura Riccardi, Federico De Biasi, Daniele Rosa-Gastaldo, Fabrizio Mancin, Marco De Vivo, Federico Rastrelli

**Affiliations:** † Molecular Modeling and Drug Discovery Lab, Istituto Italiano di Tecnologia, via Morego 30, 16163 Genova, Italy; ‡ Expert Analytics, Møllergata 8, 0179 Oslo, Norway; § Department of Chemical Sciences, University of Padova, via Marzolo 1, 35131 Padova, Italy

## Abstract

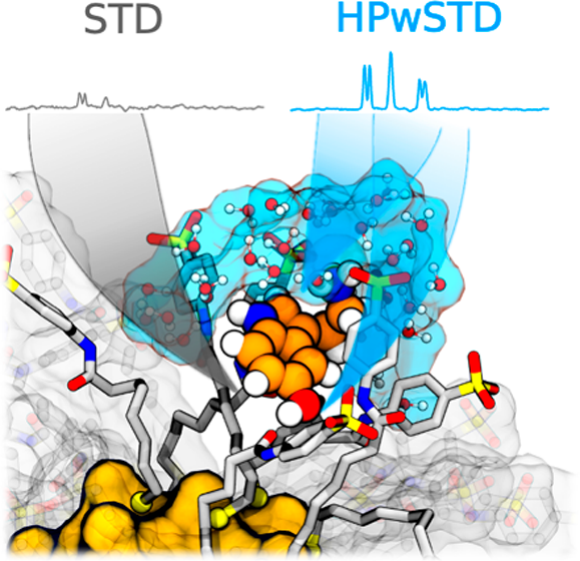

Nanoparticle-assisted nuclear magnetic resonance (NMR) chemosensing exploits monolayer-protected nanoparticles as supramolecular hosts to detect small molecules in complex mixtures via nuclear Overhauser effect experiments with detection limits down to the micromolar range. Still, the structure–sensitivity relationships at the basis of such detection limits are little understood. In this work, we integrate NMR spectroscopy and atomistic molecular dynamics simulations to examine the covariates that affect the sensitivity of different NMR chemosensing experiments [saturation transfer difference (STD), water STD, and high-power water-mediated STD]. Our results show that the intensity of the observed signals correlates with the number and duration of the spin–spin interactions between the analytes and the nanoparticles and/or between the analytes and the nanoparticles’ solvation molecules. In turn, these parameters depend on the location and dynamics of each analyte inside the monolayer. This insight will eventually facilitate the tailoring of experimental and computational setups to the analyte’s chemistry, making NMR chemosensing an even more effective technique in practical use.

^1^H nuclear magnetic resonance (NMR) spectra of organic species provide distinctive resonance patterns, allowing the unambiguous identification of the chemical species analyzed. As such, NMR spectroscopy represents an ideal technique for analyzing mixtures. However, multispecies crowding and overlapping of different resonance frequencies typically hamper the direct detection of single species in mixtures. To address this issue, we proposed a “nanoparticle-assisted NMR chemosensing” approach for the NMR identification of target analytes in complex mixtures.^1^ This method capitalizes on the reduced tumbling rates of nanoparticles and on the supramolecular hosting abilities of ligand shell-protected gold nanoparticles (AuNPs) to promote the selective magnetization/saturation transfer to the analytes and the subsequent removal of the signals from the other species.

AuNPs are particularly suited for this application because they are excellent platforms for designing macromolecular hosts, as confirmed by various proposed applications.^2−7^ The ligands constituting the AuNP’s coating form a micelle-like pseudophase that can incorporate hydrophobic guests in water (i.e., the analytes).^8,9^ Functional groups inserted into the coating ligands can provide additional or alternative interactions with the guests.^10^ The residual conformational mobility of the coating ligands can even promote the formation of transient and adaptable binding pockets with a cavitand-like structure in the monolayer.^11−13^

Several protocols can be used for nanoparticle-assisted NMR chemosensing. In early nuclear Overhauser effect (NOE) pumping experiments, magnetization was transferred from the AuNP to the analytes via a transient NOE after the signals from the fast-diffusing species (including the analyte and all of the interferents) were dephased by a diffusion filter.^6,7,14^ The modest detection limit of NOE pumping was subsequently improved by shifting to saturation transfer difference (STD) experiments. In this approach, the spin populations of the analytes interacting with the AuNPs are indirectly altered through sustained irradiation at a limited portion of the monolayer’s resonance frequencies, providing a more efficient magnetization transfer.^15−17^

More recently, we proposed a high-power water-mediated saturation transfer difference (HPwSTD) experiment^18^ and the modification uni-WASTY.^19^ In HPwSTD, the water molecules in long-lived association with the AuNP (i.e., slowly tumbling water molecules) act as additional reservoirs of saturation (Figure 1A). HPwSTD emerged as a more sensitive technique than conventional STD by decreasing the detection limit of analytes to 50 μM in reasonable acquisition times.^18^ The reasons for this remarkable performance are manifold. First, the number of slowly tumbling water molecules associated with the AuNPs is expected to be large. Second, the high-power radiofrequencies of the saturating pulses can partially saturate the AuNPs, resulting in a joint water–monolayer source of saturation. Third, the same high-power pulses can be fine-tuned to contrast the NOE contribution of the bulk water molecules surrounding the unbound analytes (particularly in uni-WASTY^19^). This NOE is generated in the fast motion regime and results in a negative polarization of the signals stemming from the unbound analytes and in the consequent reduction of the intensity of the signals produced by those analytes that are interacting with the AuNPs in water STD (wSTD) and waterLOGSY experiments.

**Figure 1:**
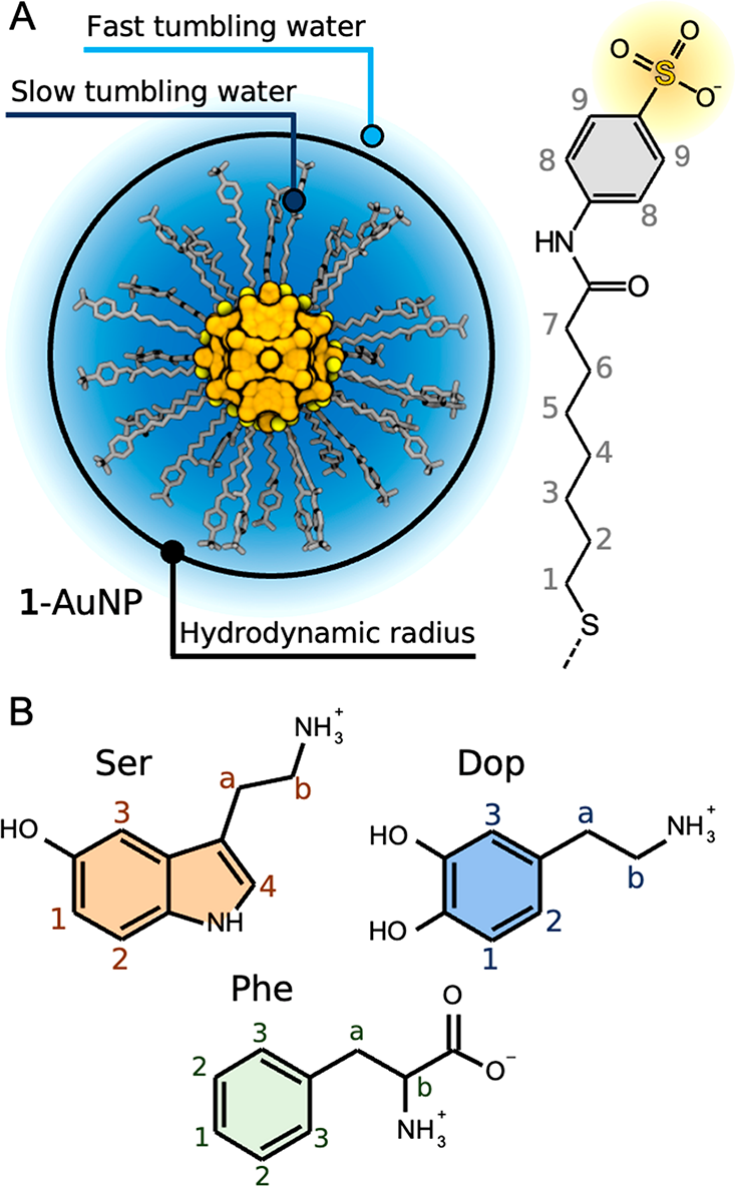
Studied systems. (A) Structure of **1**-AuNP with the formula Au_144_(SR)_60_ and the chemical structure of the anionic coating ligand. Illustration of the hydrodynamic radius of the AuNP separating slowly and quickly tumbling water molecules. (B) Chemical structures of the analytes, namely, serine (Ser), dopamine (Dop), and phenylalanine (Phe). The atom labels number the non-exchangeable, chemically equivalent hydrogen atoms.

Despite the potential of the STD-based protocols to significantly decrease the detection limit,^1,16,20^ the role of the specific interactions among the AuNPs, analytes, and solvent molecules remains poorly understood.^14,18^ On the one hand, higher affinities for an analyte should result in lower detection limits.^13,17^ However, increasing an analyte’s bound fraction leads to a severe signal broadening that restricts this approach’s applicability.^18^ On the other hand, variables such as the location of the analyte in the monolayer, its mobility, and its orientation could affect the saturation transfer efficiency and hence the sensitivity of the AuNP toward different analytes. The relevance of these parameters in relation to the detection protocol is also still unknown. Here, we report an integrated experimental and computational study investigating the chemical parameters that control the sensitivity of NMR protocols during biomarker detection.

For our investigations, we prepared two AuNP/analyte samples consisting of the same nanoreceptor, **1**-AuNP, and one of two analytes with a similar structure, namely, serotonin (Ser) and dopamine [Dop (Figure 1B)]. Ser and Dop are neurotransmitters featuring an amphiphilic cationic structure at pH 7, and they are ideal guests for **1**-AuNP, an anionic nanoparticle coated with alkylbenzenesulfonate ligands.^15,18^ The affinities of **1**-AuNP for the two analytes were determined by ^1^H NMR titrations (Figures S1–S6). In a 10 mM phosphate buffer solution, the binding constants were as follows: *K*_a_^Ser^ = (2.5 ± 0.7) × 10^3^ M^–1^ and *K*_a_^Dop^ = (8.7 ± 0.5) × 10^2^ M^–1^. These association constants were confirmed by independent DOSY experiments (Table S1).

On the basis of these data, we prepared a set of samples in which the concentration of **1**-AuNP was fixed at 0.93 μM (corresponding to an overall concentration of ligand **1** of 50 μM) and those of the analytes were 0.5 and 1.4 mM for Ser and Dop, respectively. These conditions were selected to ensure that the same amount of each analyte (14 μM, corresponding to ∼15 bound analytes per particle) was bound to **1**-AuNP. Note that the number of bound analytes per AuNP is constant for both samples, but the molar fractions of bound and unbound analytes are different (*vide infra*). We determined the raw ^1^H NMR spectrum for each sample (Figure 2A,B) followed by STD (Figure 2C,D), HPwSTD (Figure 2E,F), and wSTD (Figure 2G,H) experiments.

**Figure 2:**
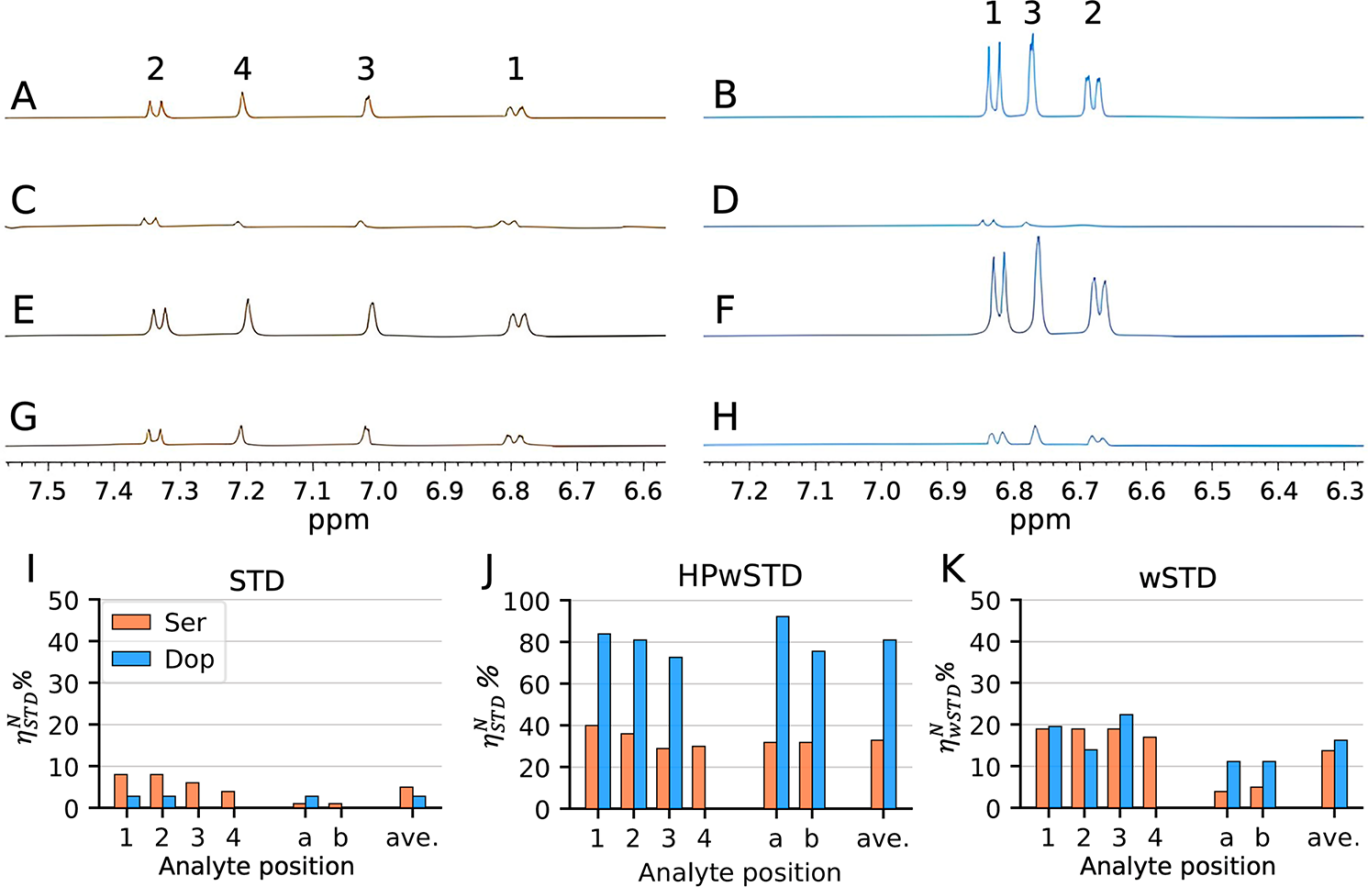
^1^H NMR spectra (500 MHz, 25 °C, 10 mM phosphate buffer, pH 7) of 0.93 μM **1**-AuNP (corresponding to a total concentration of **1** ligands of 50 μM) with (A) 0.5 mM Ser or (B) 1.4 mM Dop. (C and D) Corresponding STD NMR spectra with a 2 s saturation at 1.2 ppm. (E and F) Corresponding HPwSTD spectra with 2 s saturation by 180° Gaussian pulses (γ*B*_1_ = 750 Hz; high power) at the frequency of H_2_O. (G and H) Corresponding wSTD spectra. (I–K) Histograms of η_STD_% (or η_wSTD_%) calculated from STD, HPwSTD, and wSTD experiments, respectively, for each proton of Ser and Dop (256 scans).

When the STD experiments were performed on these samples (Figure 2C,D), the resonance frequencies of Ser were observed in the difference spectra. On the contrary, the Dop signals were significantly smaller and, in some cases, barely detectable (S/N ≈ 3). In addition, the relative signal intensities were different from those of the free species for both analytes, with signals from H_1_^Ser^/H_2_^Ser^ and H_1_^Dop^ showing an increased intensity when compared with that of the other signals from the same compound. Conversely, in the HPwSTD experiments, Ser and Dop were detected with very strong signals. Dop signals were more intense than Ser signals, and the relative intensities reproduced those of the free analytes (Figure 2E,F). Experiments were repeated with both samples complemented with phenylalanine (Phe, 0.5 mM). Notwithstanding the similarities between the chemical structures of Ser and Dop, the zwitterionic nature of Phe makes this a low-affinity species (i.e., with *K*_a_ roughly below 100 M^–1^) that does not interact significantly with **1**-AuNP under the adopted experimental conditions. Indeed, no signals of Phe were detected in the STD or HPwSTD spectra (Figures S7 and S8), confirming that the Ser and Dop signals observed in the STD spectra were due to only the AuNP–analyte interactions.

It is known in the context of epitope mapping that STD responses depend on the longitudinal relaxation times of the ligand protons. In particular, when the *T*_1_ values of the analyte protons are markedly different, STD experiments may not provide an accurate image of analyte–target interactions.^21^ On this basis, we measured the exchange-averaged *T*_1_ values in the presence of **1**-AuNP and compared them for Ser and Dop. We found that the two analytes relaxed similarly. In particular, the aromatic (i.e., H_1_ and H_3_) and aliphatic (i.e., H_a_ and H_b_) protons provided similar *T*_1_ values across Ser and Dop (Table S2), even if they are quite different from those of other protons within each molecule. Reassured by the absence of relevant relaxation differences, we determined the saturation transfer efficiency, η_STD_% (or η_wSTD_%), of each proton type as η_STD_% = 100 × (*I*_H_*n*__^off^ – *I*_H_*n*__^on^)/*I*_H_*n*__^off^, where *I*_H_*n*__^off^ and *I*_H_*n*__^on^ are the integrated signal intensities for proton H_*n*_ in the off- and on-resonance spectra, respectively.^22^

However, we noted that notwithstanding the controls and treatments described above, a direct comparison of the η_STD_% values between the two samples is still inadequate. In an STD experiment, the measured η_STD_% values are proportional to the molar fraction of the bound analyte^18^ [a similar relation holds in the case of HPwSTD (see section S5 for details)]. In our experiments, the **1**-AuNP/Ser sample contains a lower unbound molar fraction of analyte than does the **1**-AuNP/Dop sample, and consequently, the measured η_STD_% would be smaller even in the case of equal efficiency of saturation transfer. Indeed, because the concentration of the bound analyte is set to be equal, the η_STD_% measured in our samples is dependent on the total analyte concentration. We hence introduced a concentration-normalized saturation transfer efficiency, calculated as η_STD_^*N*^% *= η*_STD_% × *R*, where *R* is the ratio between the concentration of the selected analyte and the concentration of the analyte used in the smaller amount. In our case, the concentration of Ser is 0.5 mM and the concentration of Dop is 1.4 mM, so *R* = 1 for Ser and *R* = 2.8 for Dop.^18^

The normalized data reveal that Ser has average values of η_STD_^*N*^% that are larger than those of Dop in the STD experiments. In particular, the average η_STD_^*N*^% values for all of the Ser and Dop protons were 5% and 3%, respectively (Figure 2I). Interestingly, the trend was inverted in HPwSTD experiments, where there was a preference for Dop (81%) over Ser (33%) (Figure 2J). As already mentioned, HPwSTD transfers saturation both from the spins of the monolayer and from the water molecules of the solvation shell. To qualitatively distinguish these components, we also performed a low-power wSTD experiment (Figure 2G,H,K), where only the spins of water (and not those of the AuNP) are selectively saturated. As discussed above, wSTD signals result from two opposite contributions: a negative NOE produced by water molecules in long-lived association with AuNP and a positive NOE produced by bulk water molecules. The dependence of η_wSTD_% on the analyte concentration is hence more complex than in the previous cases (i.e., η_STD_%), because the presence of the NOE from bulk water reduces the saturation transfer efficiency (see section S5 for details). For this reason, the η_wSTD_^*N*^% values measured were corrected for the bulk water contribution before normalization as η_wSTD_^*N*^% = (η_wSTD_ – η_wSTD_^0^%)*R*, where η_wSTD_^0^% was measured in the absence of **1**-AuNP. The signals obtained with wSTD are more intense than those in standard STD experiments and less intense than those in HPwSTD experiments. The η_wSTD_^0^ % values for Ser and Dop are 14% and 16%, respectively, with a small preference for Dop over Ser.

Overall, the results presented above confirmed the different sensitivities of STD, wSTD, and HPwSTD experiments. They also revealed that even though AuNPs bind the same number of analyte molecules, the net response of the different NMR experiments depends on the analyte’s identity and the chemical equilibrium between its bound and unbound states. STD correctly detects Ser, while the signals for Dop are barely above the signal-to-noise ratio under the conditions employed. Instead, wSTD detects the two analytes with a similar sensitivity and a weak preference for Dop. Lastly, HPwSTD features a sensitivity much larger than that of STD, and the preference for Dop is substantially enhanced.

To obtain molecular information about these different behaviors, we used a computational approach to analyze the specific AuNP–analyte interactions. We first performed a 100 ns molecular dynamics (MD) simulation of **1**-AuNP in explicit water to equilibrate its structure. This simulation shows that the ligands extend in water to 2.5 nm from the center of mass (COM). Water molecules enter different regions of the coating monolayer at different rates [radial water exchange rate (see Figure 3A and section S1)]. At short distances from the gold center (<1.5 nm), water molecules are rarely exchanged, indicating a stable and tightly packed solvation shell. As the distance increases, the exchange of water molecules accelerates following a single-exponential function with a rate λ of 1.6 nm^–1^ until it reaches the nominal value of 58.2 nm^–2^ ns^–1^ in the bulk solvent. The hydrodynamic radius of **1**-AuNP, i.e., the distance at which the exchange rate reaches 55.1 nm^–2^ ns^–1^ (95% of the bulk value), is located at 3.0 nm [confirmed also by DOSY (see section S3)].

**Figure 3:**
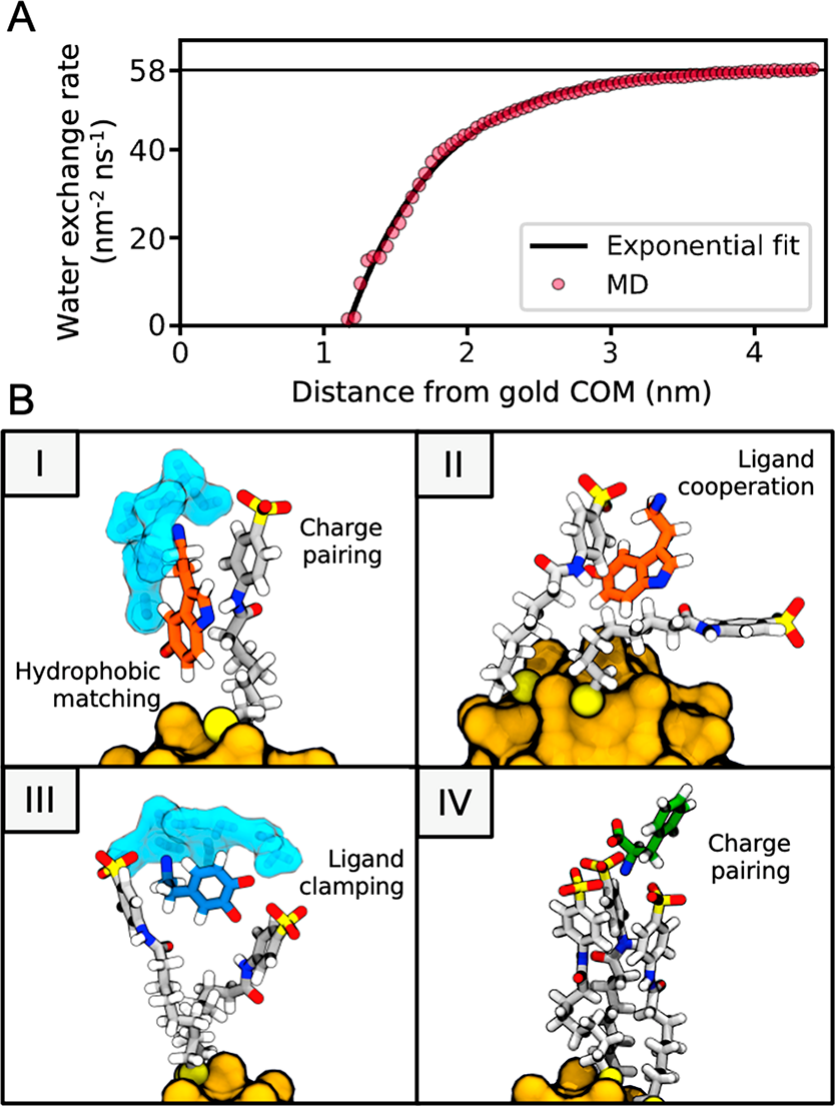
Water exchange rate and representative binding modes. (A) The radial water exchange rate is a function of the distance to the gold atoms’ center of mass (COM). The plot shows the rates calculated from MD simulations and an exponential fit. (B) Snapshots of the main modes of binding between **1**-AuNP and the analytes. Modes I and II are the predominant geometries found for Ser. Similar complexes are formed with Dop, in addition to mode III. The snapshots of binding modes I and III show the slowly tumbling water molecules in the proximity of Ser (mode I) or Dop (mode III). The interactions of Phe with the monolayer are short-lived and mainly driven by charge pairing (mode IV). Ser is shown with orange carbons, Dop with blue carbons, Phe with green carbons, and water with cyan surfaces. Ligands are shown with gray carbons. Hydrogen atoms are colored white, nitrogen atoms blue, oxygen atoms red, sulfur atoms yellow, and gold atoms mustard.

To study how the analytes interact with the AuNP’s monolayer and the surrounding water molecules, we performed three 1 μs simulations of **1-**AuNP in the presence of 10 molecules of Ser, Dop, or Phe. The average distribution of the ligands, as described by the radial distribution function (RDF), is nearly identical for the three analytes (Figure S9). All analyte molecules, including Phe, were found inside or in the vicinity of the monolayer. This result stems from the very high concentration of analytes and AuNP attained *in silico*, which favors full binding of the analytes to **1**-AuNP in the case of both high (Ser and Dop) and low (Phe) affinity. Still, this setup reproduces well the interaction conditions of the nanoparticles in the experiments (except for the negative control Phe). In fact, the number of Ser and Dop molecules included in the monolayer at any time point of the simulations (10 per nanoparticle) was similar to that of the NMR experiments (15 per nanoparticle) as calculated from the affinity constant. The full-binding conditions allowed us to obtain information about the bound states of the two analytes, which are the ones experiencing saturation transfer.

A visual inspection of the simulations identified recurrent orientations in which the analytes interact with **1**-AuNP’s monolayer (Figure 3B). In all cases, the cationic headgroup of the analytes formed an ion pair with the ligand’s anionic headgroup. The aromatic portion of the analyte was generally inserted into the monolayer to interact with either the aromatic or the aliphatic segments of the same ligand [as the one forming the ion pair with the analyte’s headgroup (mode I in Figure 3B)] or of another ligand (mode II in Figure 3B). In the case of Dop, a third binding mode was observed (mode III), where the headgroups of two ligands from **1**-AuNP simultaneously clamp the analyte, forming an ion pair with the protonated amine and an H-bond network with the catechol moiety. Lastly, in the case of Phe, we evidenced that the preferred binding with **1**-AuNP occurred through electrostatic pairing only (mode IV in Figure 3B), and that the rest of the binding modes appeared only fleetingly.

A deeper analysis of the π-stacking interactions revealed that the aromatic moieties of analytes and ligands stack mainly in a “parallel displaced” geometry (Figure S10). We also identified that Phe, the nonbinding analyte, formed significantly fewer π-stacking interactions with **1**-AuNP than its cationic counterparts (Ser and Dop). Nevertheless, Dop featured more π-stacking interactions than did Ser, suggesting that this form of interaction is not the driving factor behind STD signals.

We further characterized the specific AuNP–analyte interactions by studying the contacts responsible for the NOEs in the different NMR protocols. We computed the number of proton–proton contacts (<0.4 nm) between the analytes and the ligands, grouping all of the chemically equivalent protons (Figure 1A,B). This analysis highlighted the relevant differences between the analytes. There were 32 690 contacts for Ser, 20 012 for Dop (−39% relative to Ser), and 16 894 for Phe (−48% relative to Ser). The cumulative number of contacts decayed as a single exponential (Figure 4A). Fitting the cumulative histograms to an exponential function provided decay rates λ of 0.67, 1.04, and 1.57 ns^–1^ (section S1), which corresponded to expected lifetimes (λ^–1^) of 1.49, 0.96, and 0.64 ns for Ser, Dop, and Phe, respectively.

**Figure 4:**
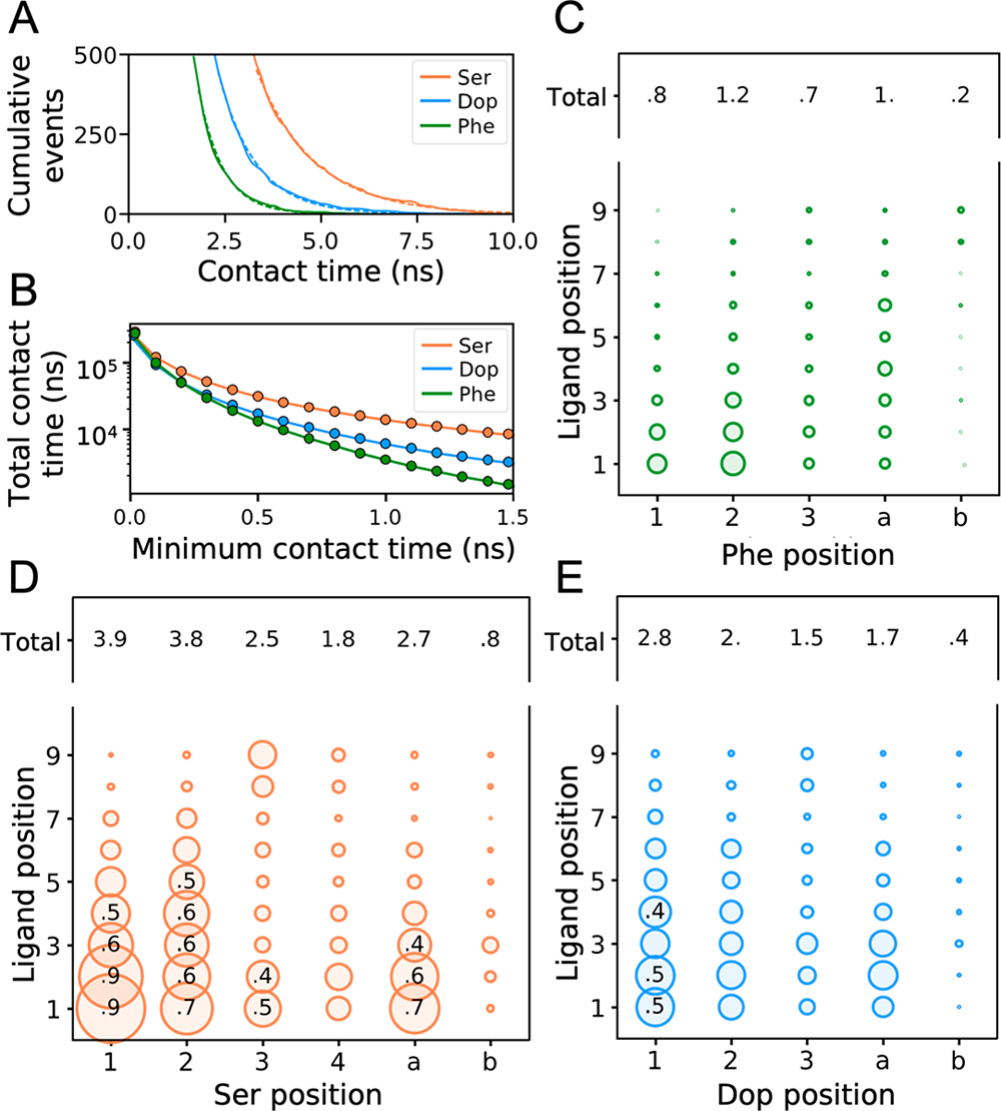
Interactions between analytes and ligands. (A) Cumulative number of contacts as a function of their lifetime. The plot shows the populations computed from MD simulations (solid lines) and their exponential fit (dashed lines). (B) Total contact time observed in each simulation as a function of the minimum contact time threshold. (C–E) Relative total contact time between the distinguishable chemical positions of the analytes and the ligands. The total contact time is normalized by the total simulation time and the number of equivalents at each pair of analyte–ligand positions. The diameter of the bubbles increases proportionally with the number of contacts. Only fractions of ≥0.4 are shown for the sake of clarity.

Note that these computations include only contacts lasting longer than 0.5 ns (i.e., 25 times the frame saving rate) to ensure precise estimates of the contact times (i.e., measurement error of ∼4%). The robustness of this threshold was assessed by calculating and plotting the total contact time as a function of the minimum contact time threshold. Figure 4B confirms that the same trend is maintained, regardless of the threshold chosen.

Hence, Ser is the analyte making the most and longest contacts with **1-**AuNP, followed by Dop and Phe. The cumulative residence times between the monolayer and the analyte (number of contacts × expected lifetime) are 48.7, 19.2, and 10.8 μs for Ser, Dop, and Phe, respectively. Accordingly, the cumulative residence time of Ser is 2.5 times larger than for Dop. This figure matches quite well the experimental results, where η_STD_^*N*^% values of Ser were 1.7 times larger than those of Dop.

Interestingly, the trends in the total number of contacts and the contact’s duration also correlate well with the analytes’ affinities (Figure 4A), suggesting that a small number of short-lived contacts indicate a weakened interaction with the monolayer. This correlation is a relevant finding because (i) it proposes a method for ranking the analytes’ affinities for AuNPs computationally and (ii) it reveals that even if the number of analytes bound to the nanoparticles is the same, their contacts with the monolayer, and consequently the saturation transfer efficiency, might differ.

Remarkably, MD simulations also allowed the explanation of the different per-proton STD signals within the same analyte. The relative contact duration between each distinguishable position of the analytes and the ligands (Figure 4C–E) discloses a conserved pattern among the analytes. The aromatic hydrogens H_1_^Ser^/H_2_^Ser^ and H_1_^Dop^ form the most contacts with the alkyl chain of the ligands (positions 1–5) compared to the other aromatic hydrogens. These positions likely identify the most hydrophobic portion of the two analytes that penetrates more deeply into the monolayer. These contact patterns are consistent with the experimental results, which showed larger values of η_STD_^*N*^% for H_1_^Ser^/H_2_^Ser^ and H_1_^Dop^ than for the rest of the signals (Figure 2I).

Subsequently, we analyzed the interactions between the analytes and the water molecules within the AuNP’s hydrodynamic radius (Figure 5), which we tentatively identified as the slowly tumbling water molecules working as saturation reservoirs in the wSTD experiments. All of the analytes formed more contacts (∼50%) with water than with the coating ligands. There were 45 167, 37 485 (−17% relative to Ser), and 20 830 (−54% relative to Ser) contacts for Ser, Dop, and Phe, respectively. The cumulative number of contacts was fitted to a single exponential (Figure 5A) to afford decay rates of 5.23, 4.80, and 14.74 ns^–1^ and expected association lifetimes of 0.19, 0.21, and 0.07 ns for Ser, Dop, and Phe, respectively. Thus, the cumulative contact times for Ser, Dop, and Phe were 8.6, 7.9, and 1.5 μs, respectively, indicating a small prevalence of Ser over Dop and a sensibly weaker association for Phe. However, when the attention is focused on the longest contacts [>1 ns (Figure 5B), i.e., the most relevant contacts in transferring the saturation from the solvent molecules^23^], the picture changes, and Dop is slightly favored with respect to Ser.

**Figure 5:**
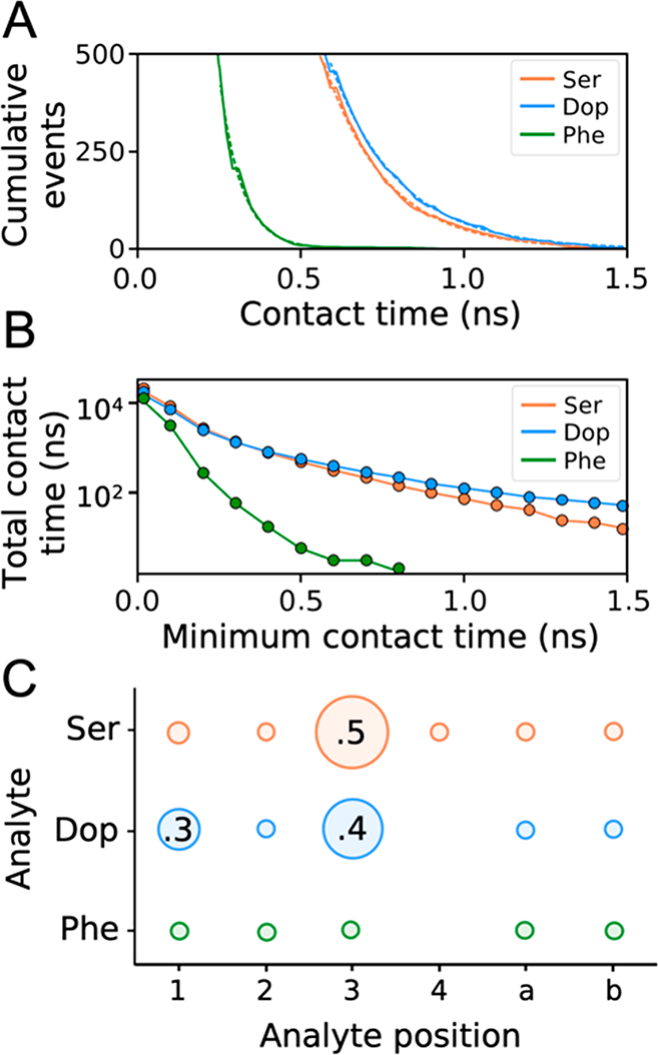
Interactions between analytes and water. (A) Cumulative number of contacts as a function of their lifetime. The plot shows the populations computed from MD simulations (solid lines) and their exponential fit (dashed lines). (B) Total contact time observed in each simulation as a function of the minimum contact time threshold. (C) Relative total contact time between the analytes’ and water’s distinguishable chemical positions. The total contact time is normalized by the total simulation time and the number of equivalents at each pair of analyte–water positions. The diameter of the bubbles increases proportionally to the number of contacts. Only fractions of ≥0.3 are shown for the sake of clarity.

The contact analysis discussed above can explain the results from wSTD. It suggests a similar ability of the nanoparticle to transfer saturation to the two analytes, with a weak preference for Dop when the most persistent contacts are considered. This result matches well with the experimental data, where η_wSTD_^*N*^% values of 14% and 16% were obtained for Ser and Dop, respectively. However, while the sensitivity trend is correctly predicted, the match between the computed contact patterns (Figure 5C) and the η_wSTD_^*N*^% values of the individual signals is poor. Our computational analysis indicates that the aromatic protons H_3_^Ser^ and H_3_^Dop^ form more contacts with water molecules than all of the other hydrogen atoms. Nevertheless, the η_wSTD_^*N*^% values for H_3_^Ser^ and H_3_^Dop^ are not different from those of all of the other spins of the analytes. It is noteworthy that the TIP3P model is thoroughly benchmarked against the AMBER family of force fields,^24,25^ and it accurately reproduces the dipole moment and dielectric constant of liquid water, which is why it was chosen in the first place. Nevertheless, the TIP3P model is also known to overestimate water’s self-diffusion coefficient,^26^ which could cause a misrepresentation of the analyte–water contacts in our simulations. Whether the discordance between experiments and simulations is also related to the use of the TIP3P water model remains to be investigated (e.g., comparing different water models).^27^

In the end, HPwSTD experiments can be examined on the basis of the results discussed so far, even if dissecting the individual contributions is not trivial, and only a qualitative analysis is possible. The first relevant information, provided by the experiments, is that the average η_STD_^*N*^% values for Ser and Dop significantly increase from 4% in STD to 15% in wSTD and 57% in HPwSTD. These figures measure the relative effectiveness of the different protocols and the respective saturation sources. The larger η_wSTD_^*N*^% values obtained with wSTD compared to the η_STD_^*N*^% values from STD confirm that the slowly tumbling water molecules are a larger and more effective source of magnetization than the monolayer’s ligands, as confirmed by calculations that indicate that the number of contacts with the solvent molecules is 2-fold larger than that of the contacts with the monolayers. Calculations also indicate that the contribution from monolayer spins favors Ser while the contribution of solvation water slightly favors Dop, and both of these suggestions are confirmed by experiments.

In the HPwSTD experiments, contacts with the nanoparticle’s monolayer and the solvation water molecules both saturate the analytes, and the negative contribution of bulk water is minimized. Stronger signals are expected, as confirmed by the larger η_STD_^*N*^% values measured with these experiments compared to those measured with STD and wSTD. Also, because (i) both saturation transfer mechanisms are enhanced in HPwSTD, (ii) solvation water molecules are a more effective saturation source, and (iii) Dop is more susceptible to gaining saturation from the solvent than Ser, the sensitivity for Dop is expected to be greater than that for Ser. Nicely, this expectation also agrees with the experimental results.

Experimental and computational results showed that Ser and Dop locate themselves in the monolayer of our anionic nanoparticle to receive saturation, albeit to different extents, from the nanoreceptor and the solvating water molecules. This behavior differs from what we recently found in a typical protein–substrate system, where the saturation was transferred to the analytes primarily through the protein’s spins.^19^ The advantage of the HPwSTD protocol hence rests in its generality, because it can exploit all of the possible saturation reservoirs without knowing the binding mode of the analyte in advance. Our data confirm that closely associated water molecules are more efficient as a saturation source than the monolayer’s spins. In addition to a sufficient affinity for the analyte, the ideal nanoparticle host should ensure good exposure of the bound analyte to solvation molecules. Accordingly, we recently showed how HPwSTD is effective even in the case of silica nanoparticles, where no monolayer contribution was possible.^20^

In this work, we compared the sensitivities of different saturation transfer NMR protocols for the nanoparticle-assisted detection of organic analytes. Experimental and computational results indicate that nanoparticle/analyte systems can behave differently by selecting the macromolecular receptor, the solvating water, or both as the main source of saturation to be transferred to the analytes. This choice depends on the binding site’s structure and the analyte’s binding pose. In this regard, MD simulations can provide precise information about the docking of the analytes to the monolayer and the specific host–guest interactions. In addition, MD contact analysis proved to be a reliable method for predicting the affinity of nanoparticles for analytes and hence explaining the sensitivity of STD experiments. These results can assist researchers in designing chemosensing experiments and virtual screening protocols attuned to the chemistry of analytes and nanoparticles of interest.

## Supporting Information Available

Materials and methods, preparation of computational models, setup of molecular dynamics simulations, NMR titration results, DOSY experiments, additional HPwSTD experiments, theoretical modeling of the NOE contribution in STD experiments, computational radial distribution functions, and π-stacking analysis (PDF)
