# Supplementary material for: Molecular Mechanisms Underlying Detection Sensitivity in Nanoparticle-Assisted NMR Chemosensing
Source: J Phys Chem Lett. 2023 Jul 27;14(30):6912–8. doi: 10.1021/acs.jpclett.3c01005 (PMC10405269; doi:10.1021/acs.jpclett.3c01005)
Supplement: Supplementary file 1 — jz3c01005_si_001.pdf [file jz3c01005_si_001.pdf]

Supplementary Information

Molecular Mechanisms Underlying Detection Sensitivity in Nanoparticle-assisted NMR Chemosensing

Sebastian Franco-Ulloa,[1,2,†] Andrea Cesari,[3,†] Laura Riccardi,[1] Federico De Biasi,[3] Daniele Rosa-Gastaldo,[3] Fabrizio Mancin, [*,3] Marco De Vivo[*,1] Federico Rastrelli,[*,3]

1. Molecular Modeling and Drug Discovery Lab, Istituto Italiano di Tecnologia, via Morego 30, 16163 Genova, Italy.
2. Expert Analytics, Møllergata 8, 0179 Oslo, Norway.
3. Department of Chemical Sciences, University of Padova, via Marzolo 1, 35131 Padova Italy.

† These authors contributed equally.

Corresponding authors:

Prof. Fabrizio Mancin – Email: fabrizio.mancin@unipd.it

Dr. Marco De Vivo – Email: marco.devivo@iit.it

Prof. Federico Rastrelli – Email: federico.rastrelli@unipd.it

Table of Contents

1. Materials and Methods 3

1.1 Reagents 3

1.2 NMR Titrations 3

1.3 Computational Models 3

1.4 Molecular Dynamics Simulations 4

2. NMR Titration 6

3. DOSY Experiments 10

4. Additional HPwSTD Experiments 12

5. Contribution to NOE in STD Experiments 14

6. Radial Distribution Functions 16

7. References 17

1. Materials and Methods

1.1 Reagents

Chemicals, including the analytes, and solvent were purchased by Merck and used as received. NMR experiments were performed on a Bruker AVANCE III spectrometer operating at 500.13 MHz ^{1}H Larmor frequency and equipped with a 5 mm z-gradient broad-band inverse (BBI) non-cryogenic probe. The STD, HPwSTD, and wSTD experiments were performed using a previously described pulse sequence.[1]

The gold nanoparticles **1**-AuNP used in all the experiments belong to a batch previously published.[2] For a detailed description of the thiol and AuNPs synthesis procedures and for a complete characterization of the nanoparticles see ref.[2]. **1**-AuNP has an average diameter of 1.6 ± 0.4 nm and an average formula $Au_{180}SR_{54}$.

1.2 NMR Titrations

The NMR titrations were performed following the shift of the analyte resonances upon subsequent additions of a 60 mM stock solution of serotonin hydrochloride or dopamine hydrochloride to a solution of **1**-AuNP (0.23 mM in thiol units) in 10 mM sodium phosphate buffer (D_2O, pD = 7.01). The titrations were fitted following a 1:1 binding model using DynaFit for Windows[18] as detailed in ref.[3]. The association constant has been calculated for each analyte resonance and the final result is the average of these values. On the basis of our previous experience,[2] we considered that on average the binding sites on the nanoparticles are composed of two thiols (i.e., 26 binding sites per nanoparticle). In addition, the use of a 1:1 binding model rests upon the assumption that each binding site is independent and, as a consequence, the binding constants obtained must be considered as an average estimate.

1.3 Computational Models

The partial charges of the thiols (**1**-AuNP) and the analytes (Ser, Dop, and Phe) were calculated with the RESP fitting procedure as implemented in the R.E.D. server.[4] The initial conformation and the parameter files for **1**-AuNP were generated with the NanoModeler webserver.[5,6] The parameters of the analytes were taken from the GAFF force field.[7]

1.4 Molecular Dynamics Simulations

For the molecular dynamics (MD) simulation of **1**-AuNP alone in water, one AuNP was placed in a dodecahedral box, leaving a minimum distance of 2.2 nm between the nanoparticle and the box's edges. The box was filled with water molecules (TIP3P model[8]) and enough sodium ions were added to neutralize the system.[9] The system was minimized using the steepest descent method. The MD run started with a 500 ps-long simulation in the NVT statistical ensemble, using the V-rescale thermostat (τ = 0.1 ps, T = 310 K). Then, the system was pressurized with a 500 ps-long simulation in the NPT statistical ensemble, adding a Berendsen barostat (τ = 2 ps, $\kappa = 4.5 \times 10^{-5}$ bar^{-1}, P = 1 bar).[10] After the equilibration, the system was simulated for 100 ns using a Parrinello-Rahman barostat (τ = 2 ps, $\kappa = 4.5 \times 10^{-5}$ bar^{-1}, P = 1 bar).[11] We used a timestep of 2 fs, truncated short-range nonbonded interactions at 1.2 nm, and accounted for long-range electrostatics with the PME method of fourth order.[12] Frames were saved every 20 ps. All bonds were constrained using the LINCS algorithm.[13] All simulations were run in Gromacs-v2020.2[14] and analyzed with in-house scripts using the MDAnalysis Python library.[15]

To calculate the water exchange rate, we first defined discrete shells around the AuNP (i.e., binning the distance to the gold atoms' center of mass, COM) and calculated the number of water molecules entering each shell at each timestep. The mean number of exchanged water molecules was then normalized by the density of the solvent at each shell and the increasing size of the shells at longer distances. Thus, the water exchange rate Ξ was calculated with **Equation S1**, where $\xi(r)$ is the number of water molecules entering the shell at a distance r, $N(r)$ is the total number of water molecules in that shell, R is the distance to the outermost shell (set to 4.5 nm), N_T is the total number of water molecules within R, δr is the shell width (set to 4.5×10^{-2} nm), Δt is the time between saved frames, and $\langle \cdot \rangle$ indicates a time average.

$$\Xi(r) = \left[\frac{3N_T}{4\pi R^3 \Delta t \langle N(r) \rangle} \delta r\right] \langle \xi(r) \rangle \qquad \textbf{(S1)}$$

The radial distribution function (RDF) of unit A with respect to the gold atom's COM was computed using **Equation S2**, where δ_D is Dirac's Delta function, and N_A is the number of atoms in unit A.

$$G_{AB}(r) = \frac{1}{N_A}\sum_{i=1}^{N_A}\langle\delta_D(r_i - r)\rangle \quad \textbf{(S2)}$$

For the simulations in the presence of analytes, one **1**-AuNP was placed in a dodecahedral box, leaving a minimum distance of 1.4 nm between the nanoparticle and the box's edges. Then, ten copies of an analyte (Ser, Dop, or Phe) were randomly inserted in the simulation box. Each system was solvated, neutralized, minimized, thermalized, and pressurized as described above. These systems were simulated for 1 μs, each. The resulting trajectories were used to analyze the close contacts formed with the analytes. For this, we computed the distance between all the protons of the analytes and all the protons of the AuNP, and we classified the proton-proton pair as a contacting event if their distance was shorter than 0.4 nm. Then, we tracked the number of consecutive frames in which the same contact was present. For this analysis, we considered each residue and chemical position independently, i.e., two protons in the same residue were considered identical if they were not distinguishable from ^{1}H-NMR spectroscopy (**Figure 1** in the main text). For precision purposes, we discarded the contacts shorter than 0.5 ns long (i.e., 25 times the saving timestep, see above).

The duration of the proton-proton contacts was quantified by first computing the cumulative number of events at different residence times. Then, an exponential distribution (**Equation S3**) was fit to the long-lasting regime (i.e., points with populations of 1000 or less). The parameter λ reflects how fast the number of contacts decays with their lifetime, and λ^{-1} is the expected value of the exponential distribution fitted.

$$n(t) = n_0 e^{-\lambda t} \quad \textbf{(S3)}$$

2. NMR Titration

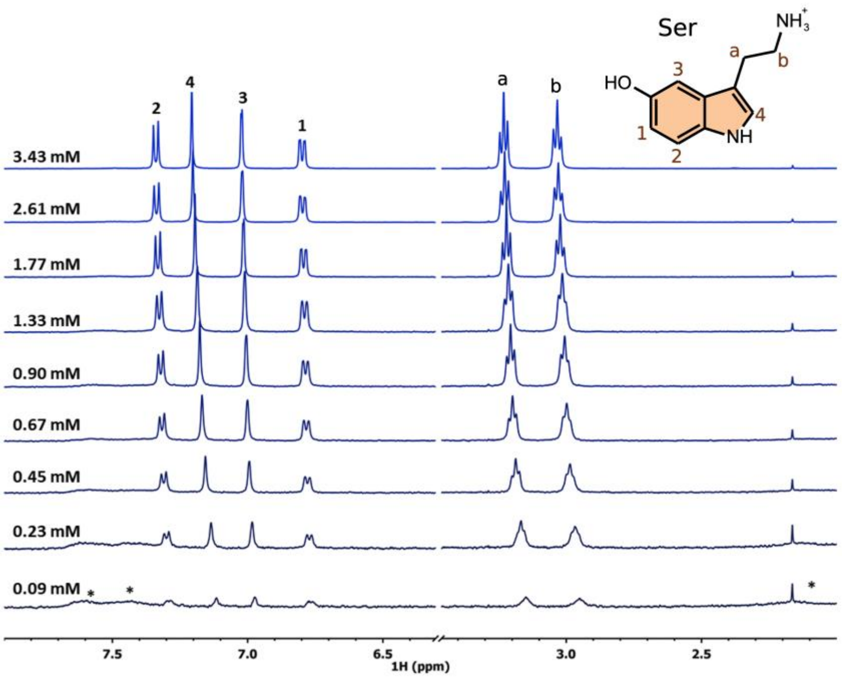

Figure S1. NMR titration of **1**-AuNP with serotonin hydrochloride in phosphate buffer (D_2O, pD = 7.01, T = 25 °C). The asterisks denote the **1**-AuNP resonances. The spectral region containing the H_2O signal at 4.6 ppm was removed for clarity.

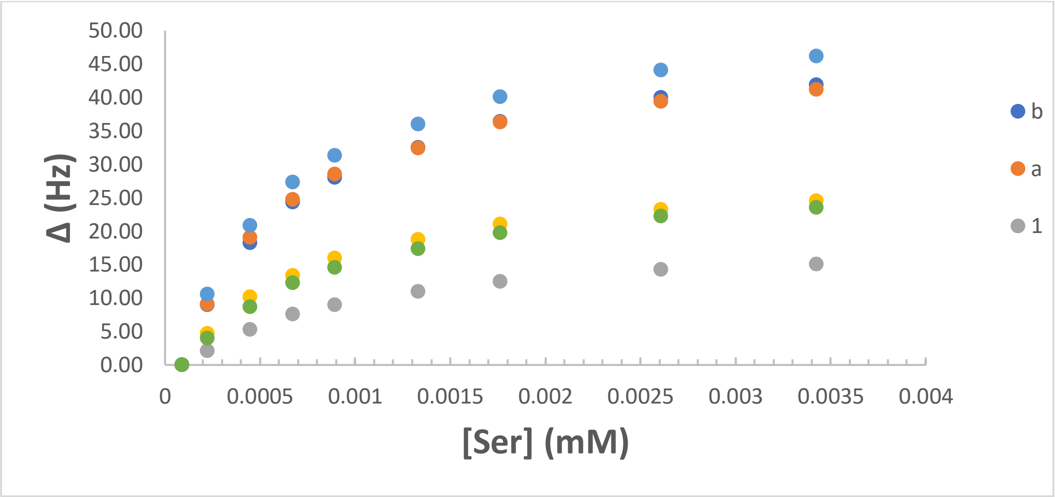

Figure S2. Plot of the frequency shifts versus the serotonin concentration for each serotonin NMR signal obtained from the NMR titration reported in **Figure S1**.

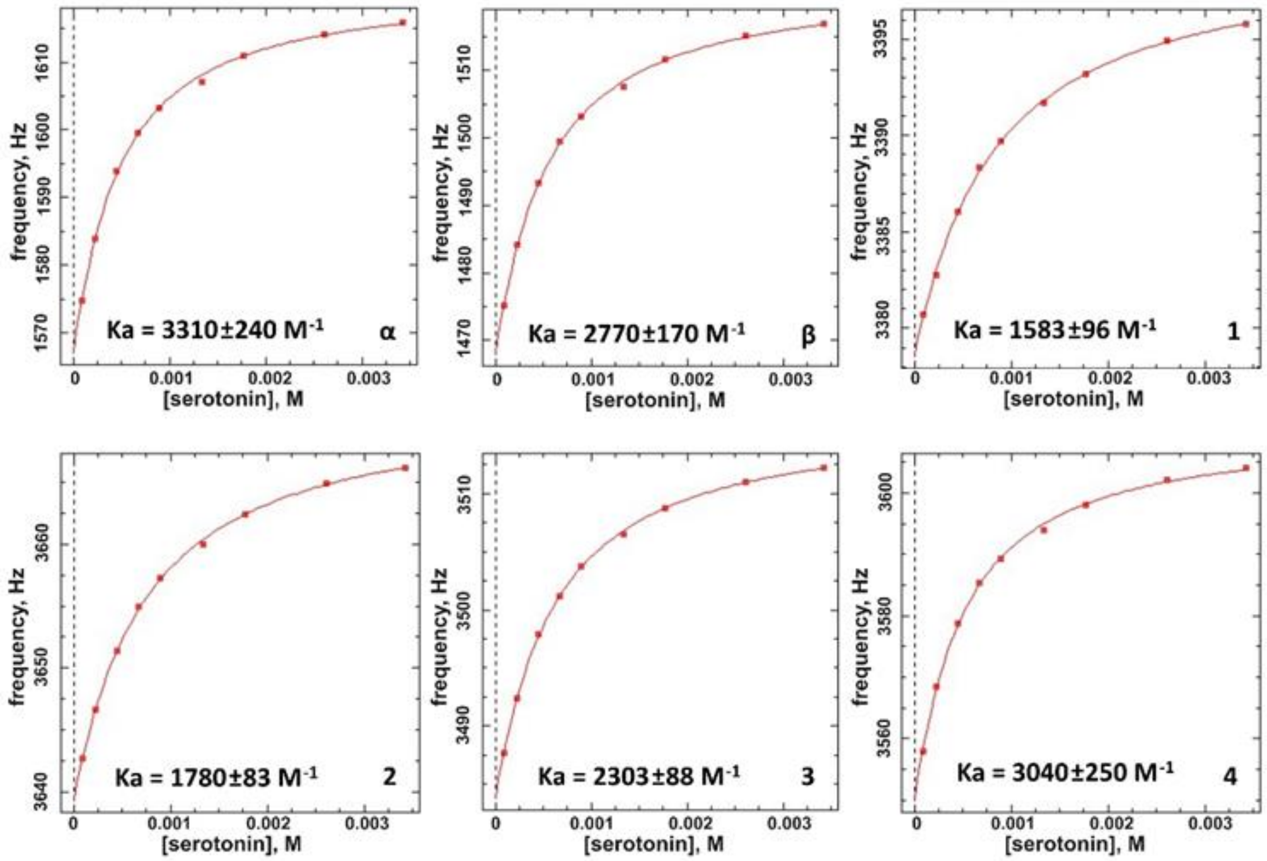

Figure S3. Plot of the fitting results and binding constant calculated for each serotonin signal. The average binding constant is $K_a^{Ser} = (2.46 \pm 0.69) \times 10^3\ M^{-1}$.

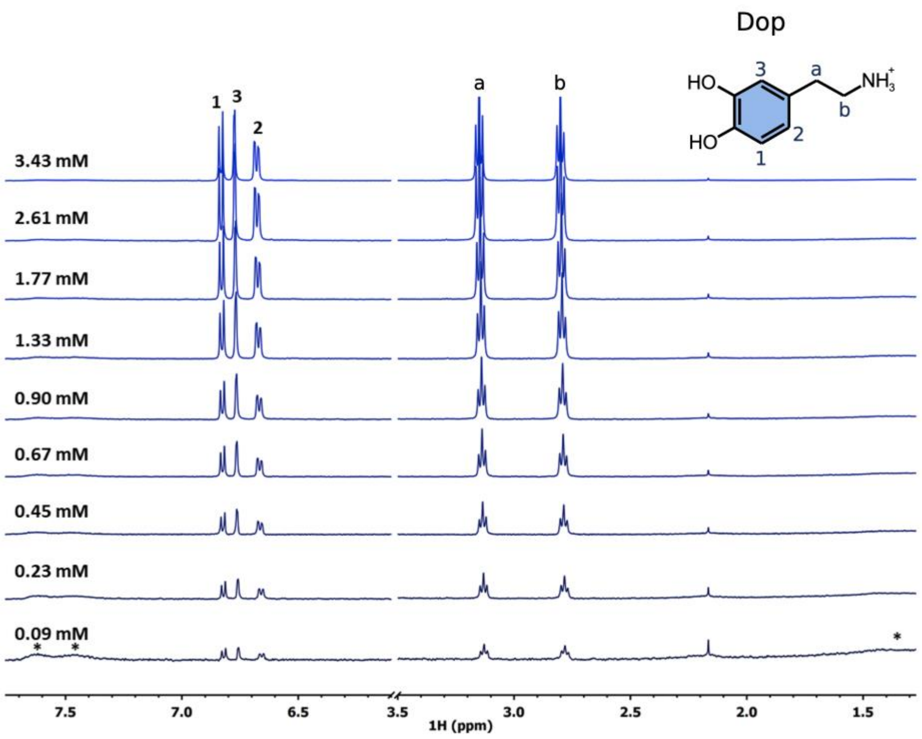

Figure S4. NMR titration of **1**-AuNP with dopamine hydrochloride in phosphate buffer (D_2O, pD = 7.01, T = 25 °C). The asterisks denote the **1**-AuNP resonances. The spectral region containing the H_2O signal at 4.6 ppm was removed for clarity.

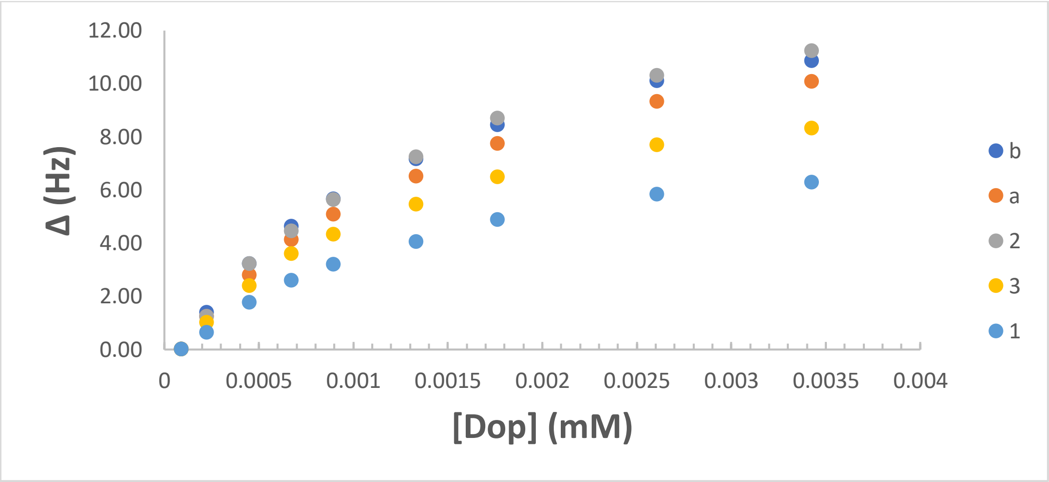

Figure S5. Plot of the frequency shifts versus the dopamine concentration for each dopamine NMR signal obtained from the NMR titration reported in **Figure S4**.

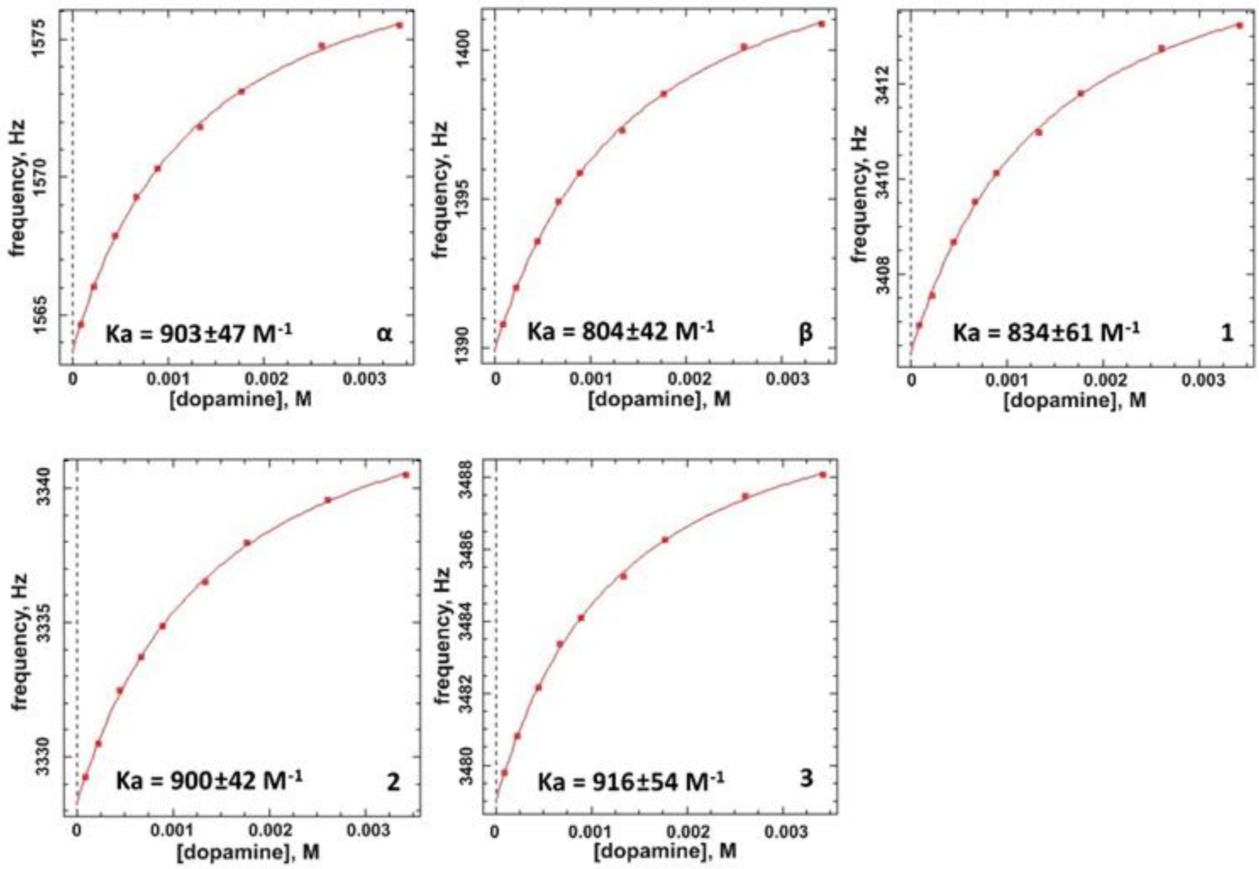

Figure S6. Plot of the fitting results and binding constant calculated for each dopamine signal. The average binding constant is $K_a^{Dop} = (0.87 \pm 0.05) \times 10^3\ M^{-1}$.

3. DOSY Experiments

DOSY experiments were performed on samples containing 9.3 μM **1**-AuNP and 0.5 mM Ser or 1.4 mM Dop, respectively. Concentration of the analytes' fractions bound to the nanoparticles was calculated from their apparent diffusion coefficients according to **Equation S4**[16]:

$$\chi_{hg} = \frac{D_{guest} - D_{obs}}{D_{guest} - D_{host/guest}} \qquad \textbf{(S4)}$$

Using the analyte bound molar fraction was possible also to calculate the association constants by a single measurement determination.[17]

Table S1. Relevant parameters of the DOSY experiments (D_2O, 25 °C, HEPES buffer 10 mM, pH=7) for Ser (**1**-AuNP 9.3 μM + Ser 0.5 mM) mixtures and Dop (**1**-AuNP 9.3 μM + Dop 1.4 mM).

	D_g-D_{obs} (m^2s^{-1})	D_g-D_{hg} (m^2s^{-1})	χ_{hg}	$[\text{Analyte}]_{bound}$ (M)	K_a (M^{-1})
Ser	$(2.3 \pm 0.3) \times 10^{-10}$	$(5.3 \pm 0.5) \times 10^{-10}$	0.44 ± 0.09	$(22 \pm 5) \times 10^{-5}$	$(2.8 \pm 1.7) \times 10^{3}$
Dop	$(1.2 \pm 0.1) \times 10^{-10}$	$(5.8 \pm 0.3) \times 10^{-10}$	0.20 ± 0.03	$(28 \pm 4) \times 10^{-5}$	$(1.1 \pm 0.4) \times 10^{3}$

On the contrary, the diffusion coefficient of pure Phe ([Phe] = 1.4 mM, D = [6.2 ± 0.1] x 10^{-10} m^2 s^{-1}) was not affected by the presence of the nanoparticle [**1**-AuNP] = 0.93 μM (D = [6.20 ± 0.01] x 10^{-10} m^2 s^{-1}).

The hydrodynamic radius of r_H = 4.2 ± 2.3 nm, calculated from DOSY experiment was in agreement with that found using molecular dynamics analysis. This radius was expressed by means of the Stokes–Einstein equation: $D = k_bT/(6\pi\eta r_H)$, where k_b is the Boltzmann constant, T is the absolute temperature, and η the viscosity of the medium.[16]

Table S2. T_1 relaxation measurements (500 MHz, 25 °C, phosphate buffer 10 mM, pH = 7) of 0.93 μM **1**-AuNP (corresponding to a total concentration of **1** ligand of 50 μM) with 0.5 mM Ser or 1.4 mM Dop.

Ser/**1**-AuNP	T_1 (s)	Error (s)	Dop/**1**-AuNP	T_1 (s)	Error (s)
H_1	3.39	0.07	H_1	3.50	0.01
H_2	3.17	0.06	H_2	2.49	0.01
H_3	3.18	0.04	H_3	3.61	0.01
H_4	3.85	0.04			
CH_{2a}	1.11	0.01	CH_{2a}	1.09	<0.01
CH_{2b}	0.97	0.02	CH_{2b}	0.97	<0.01

4. Additional HPwSTD Experiments

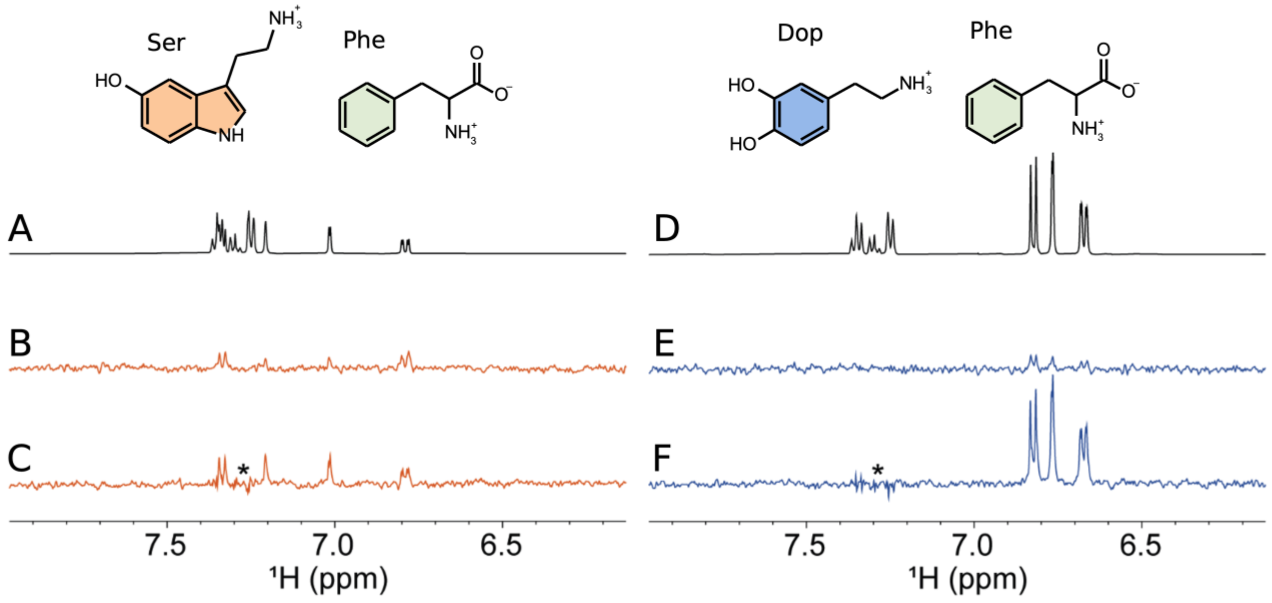

Figure S7. A. ^{1}H NMR spectrum of 0.93 µM **1**-AuNP (corresponding to 50 µM thiol **1**) with 0.5 mM Ser and 0.5 mM Phe. **B.** STD NMR spectrum with 2 s saturation at 1.2 ppm. **C.** Water-STD spectrum with 2 s saturation by 180° Gaussian pulses (γB_1 = 750 Hz, high power) at the frequency of H_2O. **D.** ^{1}H NMR spectrum of 0.93 µM **1**-AuNP (corresponding to 50 µM thiol **1**) with 1.4 mM Dop and 0.5 mM Phe. **E.** STD NMR spectrum with 2 s saturation at 1.2 ppm. **F.** Water-STD spectrum with 2 s saturation by 180° Gaussian pulses (γB_1 = 750 Hz, high power) at the frequency of H_2O. Asterisks denote cancellation artifacts.

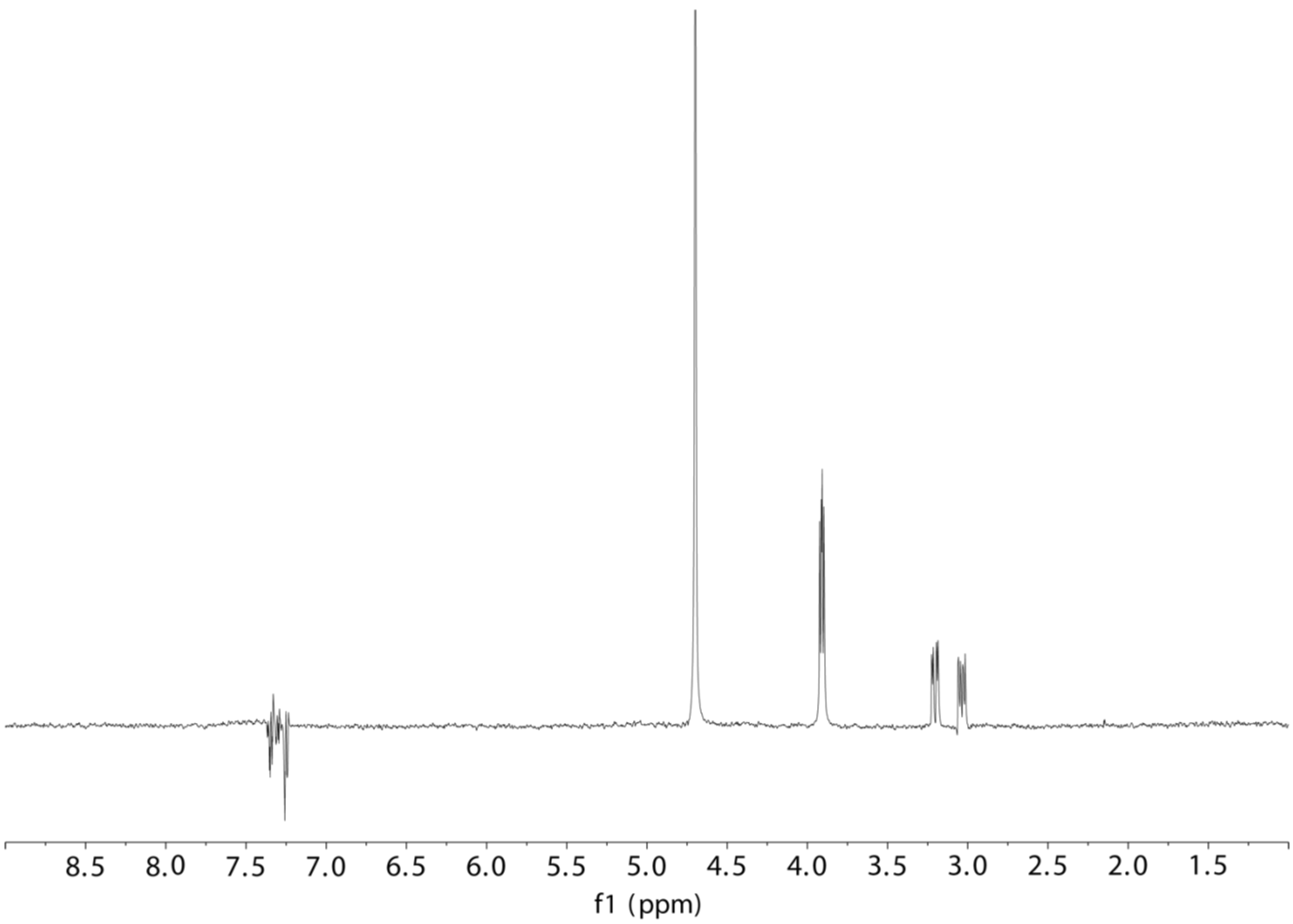

Figure S8. HPwSTD of Phe alone in the presence of **1**-AuNPs. Signals of the aromatic moiety are canceled, those in the aliphatic region appear as false positives because of the radiofrequency "spillover effect" discussed in ref[18].

5. Contribution to NOE in STD Experiments

To rationalize the different contributions affecting spin population of a guest in binding equilibrium with a host, we considered a simplified model composed by two spins I and S located in the guest (G) and in the host (H) molecule, respectively. Both these spins can be found in the free (I_f, S_f) and in the bound (I_b, S_b) state. All the contributions to NOE are depicted in **Scheme S1**. The contribution arising from water spins is active only in water-mediated experiments, i.e., HPwSTD and wSTD.

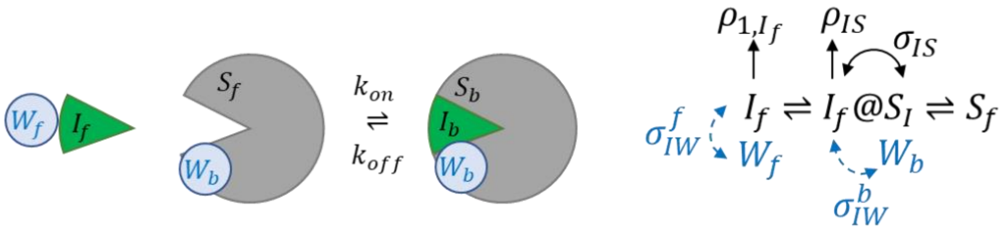

Scheme S1. Model system for a host–guest equilibrium. The guest (green) and the host (grey) contain only spins I and S respectively, and the cross-relaxation σ_{IS} is active just during the binding events. Free and bound states are denoted as "f" and "b." H_2O spins are represented by W.

In **Scheme S1**, k_{on} and k_{off} are the kinetic constants of the formation and dissociation of the complex. We have already shown that in the fast chemical exchange regime, where both $k_{on}[H]$ and k_{off} are much larger than the NMR relaxation rates, the total NOE enhancement in the case of a single signal observed for I_f and I_b is given by **Equation S5**[1]:

$$\eta = \frac{[G@H]\sigma_{IS}}{[G]\rho_{1,I_f} + [G@H]\rho_{IS}} \qquad \textbf{(S5)}$$

where $[G]$ and $[G@H]$ are the concentrations of non-bound and bound guest, respectively, ρ_{1,I_f} represents the longitudinal relaxation rate constant for I_f, σ_{IS}, and ρ_{IS} are the longitudinal relaxation rate in

the bound state and cross-relaxation rate constants involved in the dipolar interaction responsible for the saturation transfer, respectively (**Scheme S1**).

If the $[G@H]$ is negligible with respect to $[G]_{tot}$ and $\rho_{IS} \approx \rho_{1,I_f}$, then $[G]\rho_{1,I_f} + [G@H]\rho_{IS} \approx [G]\rho_{1,I_f}$ and $[G] \approx [G]_{tot}$. Accordingly, we can approximate **Equation S5** as **Equation S6**:

$$\eta \approx \frac{[G@H]\sigma_{IS}}{[G]_{tot}\rho_{1,If}} \qquad \textbf{(S6)}$$

Hence, the NOE enhancement is proportional to the fraction of guest bound to the nanoparticles or inversely proportional to the total guest concentration if $[G@H]$ is fixed (as in the case of the experiments in **Figure 2** in the main text).

A similar **Equation S7** can be derived for the HPwSTD experiments, taking into account that the analyte receives saturation both form the solvation water and the monolayer:

$$\eta \approx \frac{[G@H](\sigma_{IS} + \sigma_{IW}^{b})}{[G]_{tot}\rho_{1,If}} \qquad \textbf{(S7)}$$

where σ_{IW}^{b} is the cross-relaxation rate constant between I_b and the water molecules in long-lived association with the nanoparticle W_b.

In the case of wSTD the total NOE enhancement must take into account also the contribution of water spins in free (W_f) and bound (W_b) state. According to Dalvit and co-workers the two effects are proportional to the fraction of bound and free analytes.[19] Consequently, **Equation S5** can turn into **Equation S8**:

$$\eta \approx \frac{[G@H]\left(\sigma_{IS} + \sigma_{IW}^{b}\right) - [G]\sigma_{IW}^{f}}{[G]\rho_{1,If} + [G@H]\rho_{IS}} \approx \frac{[G@H](\sigma_{IS} + \sigma_{IW}^{b})}{[G]\rho_{1,If} + [G@H]\rho_{IS}} - \frac{[G]\sigma_{IW}^{f}}{[G]\rho_{1,If} + [G@H]\rho_{IS}} \qquad \textbf{(S8)}$$

where σ_{IW}^{f} is the cross-relaxation rate constant between I_f and the bulk water molecules. By applying the same approximations as above for a sample where the fraction of bound analyte is small ($[G]\rho_{1,I_f} + [G@H]\rho_{IS} \approx [G]\rho_{1,I_f}$ and $[G] \approx [G]_{tot}$), we obtain **Equation S9**.

$$\eta \approx \frac{[G@H](\sigma_{IS} + \sigma_{IW}^{b})}{[G]_{tot}\rho_{1,If}} - \frac{[G]_{tot}\sigma_{IW}^{f}}{[G]_{tot}\rho_{1,If}} \approx \frac{[G@H](\sigma_{IS} + \sigma_{IW}^{b})}{[G]_{tot}\rho_{1,If}} - \frac{\sigma_{IW}^{f}}{\rho_{1,If}} \quad \textbf{(S9)}$$

6. Radial Distribution Functions

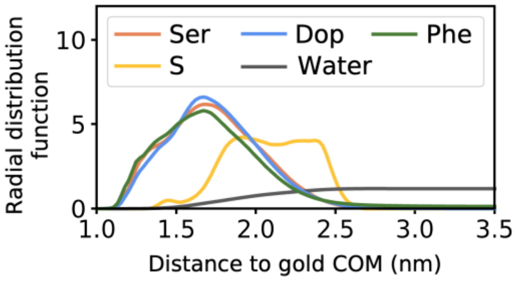

Figure S9. Radial distribution function (RDF) of the analytes' COM, from their respective simulations, with respect to the distance to the gold core's COM. The plot also shows the RDF of the solvent and the ligands' terminal sulfur atom computed from the simulation with Ser.

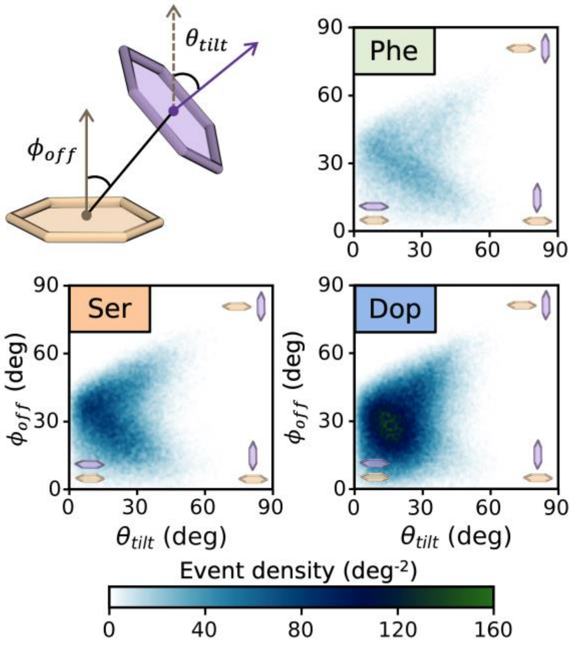

Figure S10. Stacking interactions between the analytes and the ligands. The top left panel defines the tilt angle θ_{tilt} and the offset angle ϕ_{off}. θ_{tilt} is the angle formed between the aromatic rings' plane. ϕ_{off} is the angle between a vector normal to one of the aromatic rings and the vector connecting both rings' centroids. The density maps show the geometry of the π-stacking interactions when the distance between the rings' centroids is less than 0.4 nm.

7. References

(1) De Biasi, F.; Rosa-Gastaldo, D.; Sun, X.; Mancin, F.; Rastrelli, F. Nanoparticle-Assisted NMR Spectroscopy: Enhanced Detection of Analytes by Water-Mediated Saturation Transfer. *J Am Chem Soc* **2019**, *141* (12), 4870–4877. https://doi.org/10.1021/jacs.8b13225.

(2) Gabrielli, L.; Rosa-Gastaldo, D.; Salvia, M. V.; Springhetti, S.; Rastrelli, F.; Mancin, F. Detection and Identification of Designer Drugs by Nanoparticle-Based NMR Chemosensing. *Chem Sci* **2018**, *9* (21), 4777–4784. https://doi.org/10.1039/c8sc01283k.

(3) De Biasi, F.; Rosa-Gastaldo, D.; Mancin, F.; Rastrelli, F. Hybrid Nanoreceptors for High Sensitivity Detection of Small Molecules by NMR Chemosensing. *Chemical Communications* **2021**, *57* (24), 3002–3005. https://doi.org/10.1039/D0CC07559K.

(4) Vanquelef, E.; Simon, S.; Marquant, G.; Garcia, E.; Klimerak, G.; Delepine, J. C.; Cieplak, P.; Dupradeau, F. Y. R.E.D. Server: A Web Service for Deriving RESP and ESP Charges and Building Force Field Libraries for New Molecules and Molecular Fragments. *Nucleic Acids Res* **2011**, *39* (SUPPL. 2), W511–W517. https://doi.org/10.1093/nar/gkr288.

(5) Franco-Ulloa, S.; Riccardi, L.; Rimembrana, F.; Pini, M.; De Vivo, M. NanoModeler: A Webserver for Molecular Simulations and Engineering of Nanoparticles. *J Chem Theory Comput 15* (3), 2022–2032. https://doi.org/10.1021/acs.jctc.8b01304.

(6) Franco-Ulloa, S.; Riccardi, L.; Rimembrana, F.; Grottin, E.; Pini, M.; De Vivo, M. NanoModeler CG: A Tool for Modeling and Engineering Functional Nanoparticles at a Coarse-Grained Resolution. *J. Chem. Theory Comput* **2023**, *19*, 1582–1591. https://doi.org/10.1021/acs.jctc.2c01029.

(7) Wang, J.; Wolf, R. M.; Caldwell, J. W.; Kollman, P. A.; Case, D. A. Development and Testing of a General Amber Force Field. *J Comput Chem* **2004**, *25* (9), 1157–1174. https://doi.org/10.1002/jcc.20035.

(8) Jorgensen, W. L.; Chandrasekhar, J.; Madura, J. D.; Impey, R. W.; Klein, M. L. Comparison of Simple Potential Functions for Simulating Liquid Water. *J Chem Phys* **1983**, *79* (2), 926–935. https://doi.org/10.1063/1.445869.

(9) Chen, A. A.; Pappu, R. V. Parameters of Monovalent Ions in the AMBER-99 Forcefield: Assessment of Inaccuracies and Proposed Improvements. *Journal of Physical Chemistry B* **2007**, *111* (41), 11884–11887. https://doi.org/10.1021/jp0765392.

(10) Berendsen, H. J. C.; Postma, J. P. M.; van Gunsteren, W. F.; Dinola, A.; Haak, J. R. Molecular Dynamics with Coupling to an External Bath. *J Chem Phys* **1984**, *81* (8), 3684–3690. https://doi.org/10.1063/1.448118.

(11) Parrinello, M.; Rahman, A. Polymorphic Transitions in Single Crystals: A New Molecular Dynamics Method. *J Appl Phys* **1981**, *52* (12), 7182–7190. https://doi.org/10.1063/1.328693.

(12) Darden, T.; York, D.; Pedersen, L. Particle Mesh Ewald: An N·log(N) Method for Ewald Sums in Large Systems. *J Chem Phys* **1993**, *98* (12), 10089–10092. https://doi.org/10.1063/1.464397.

(13) Hess, B.; Bekker, H.; Berendsen, H. J. C.; Fraaije, J. G. E. M. LINCS: A Linear Constraint Solver for Molecular Simulations. *J Comput Chem* **1997**, *18* (12), 1463–1472. https://doi.org/10.1002/(SICI)1096-987X(199709)18:12<1463::AID-JCC4>3.0.CO;2-H.

(14) Hess, B.; Kutzner, C.; van der Spoel, D.; Lindahl, E. GROMACS 4: Algorithms for Highly Efficient, Load-Balanced, and Scalable Molecular Simulation. *J Chem Theory Comput* **2008**, *4* (3), 435–447. https://doi.org/10.1021/ct700301q.

(15) Michaud-Agrawal, N.; Denning, E. J.; Woolf, T. B.; Beckstein, O. MDAnalysis: A Toolkit for the Analysis of Molecular Dynamics Simulations. *J Comput Chem* **2011**, *32* (10), 2319–2327. https://doi.org/10.1002/jcc.21787.

(16) Pagès, G.; Gilard, V.; Martino, R.; Malet-Martino, M. Pulsed-Field Gradient Nuclear Magnetic Resonance Measurements (PFG NMR) for Diffusion Ordered Spectroscopy (DOSY) Mapping. *Analyst* **2017**, *142* (20), 3771–3796. https://doi.org/10.1039/C7AN01031A.

(17) Cameron, K. S.; Fielding, L. NMR Diffusion Spectroscopy as a Measure of Host−Guest Complex Association Constants and as a Probe of Complex Size. *J Org Chem* **2001**, *66* (21), 6891–6895. https://doi.org/10.1021/jo010081x.

(18) De Biasi, F.; Mascitti, B. B.; Kupče, Ē.; Rastrelli, F. Uniform Water-Mediated Saturation Transfer: A Sensitivity-Improved Alternative to WaterLOGSY. *Journal of Magnetic Resonance* **2022**, *338*, 107190. https://doi.org/10.1016/j.jmr.2022.107190.

(19) Dalvit, C.; Fogliatto, G.; Stewart, A.; Veronesi, M.; Stockman, B. WaterLOGSY as a Method for Primary NMR Screening: Practical Aspects and Range of Applicability. *J Biomol NMR* **2001**, *21* (4), 349–359. https://doi.org/10.1023/A:1013302231549.
